# Italian intersocietal recommendations for restructuring the diagnostic-therapeutic pathway for the implementation and appropriate use of anti-amyloid monoclonal antibodies in Alzheimer’s disease

**DOI:** 10.1007/s10072-025-08576-y

**Published:** 2025-10-17

**Authors:** Alberto Benussi, Federica Agosta, Alba Rosa Alfano, Antonio Antico, Giuseppe Bellelli, Laura Bonanni, Gabriella Bottini, Marco Bozzali, Ovidio Brignoli, Giuseppe Bruno, Annachiara Cagnin, Sonia Francesca Calloni, Diego Cecchin, Marcello Ciaccio, Sirio Cocozza, Mirco Cosottini, Diego De Leo, Andrea Falini, Lorenzo Gaetani, Fabio Gotta, Maria Infantino, Raffaele Lodi, Giancarlo Logroscino, Elena Marcello, Camillo Marra, Walter Marrocco, Patrizia Mecocci, Enrico Mossello, Alessandro Padovani, Lorenzo Palleschi, Leonardo Pantoni, Lucilla Parnetti, Sandro Sorbi, Alessandro Tessitore, Andrea Ungar

**Affiliations:** 1 Neurology Unit, Department of Medical, Surgical and Health Sciences, University of Trieste, Strada di Fiume, 447, 34149 Trieste, Italy; 2 Neurology Clinic, Department of Medicine, Surgery and Health Sciences, Azienda Sanitaria Universitaria Giuliano Isontina, Trieste, Italy; 3 Neuroimaging Research Unit, Division of Neuroscience, IRCCS San Raffaele Scientific Institute, Milan, Italy; 4 Neurology Unit, IRCCS San Raffaele Scientific Institute, Milan, Italy; 5 Vita-Salute San Raffaele University, Milan, Italy; 6 Department of Internal Medicine and Medical Specialties, UOC Geriatrics, Sapienza University of Rome, Rome, Italy; 7 Department of Laboratory Medicine, Hospital “Ca’ Foncello”, Treviso, Italy; 8 School of Medicine and Surgery, University of Milano-Bicocca, Milan, Italy; 9 Acute Geriatric Unit, Fondazione IRCCS San Gerardo dei Tintori, Monza, Italy; 10 Department of Medicine and Aging Sciences, “G. d’Annunzio” University of Chieti-Pescara, Chieti, Italy; 11 Department of Brain and Behavioral Sciences, University of Pavia, Pavia, Italy; 12 Cognitive Neuropsychology Centre, ASST Grande Ospedale Metropolitano Niguarda, Milan, Italy; 13 NeuroMi – Milan Center for Neuroscience, Milan, Italy; 14 “Rita Levi-Montalcini” Department of Neuroscience, University of Torino, Turin, Italy; 15 Neurology Unit 2U, Azienda Ospedaliero-Universitaria Città della Salute e della Scienza di Torino, Turin, Italy; 16 Italian College of General Practitioners and Primary Care Professionals (SIMG), Florence, Italy; 17 Department of Human Neuroscience, Sapienza University of Rome, Rome, Italy; 18 Department of Neuroscience, University of Padova, Padova, Italy; 19 Padua Neuroscience Center, University of Padova, Padova, Italy; 20 Neuroradiology Unit & CERMAC, IRCCS Ospedale San Raffaele, Milan, Italy; 21 Nuclear Medicine Unit, Department of Medicine (DIMED), University of Padova, Padova, Italy; 22 Department of Biomedicine, Neurosciences and Advanced Diagnostics, University of Palermo, Palermo, Italy; 23 Department of Laboratory Medicine, University Hospital “Paolo Giaccone”, Palermo, Italy; 24 Department of Advanced Biomedical Sciences, University “Federico II”, Naples, Italy; 25 Neuroradiology Unit, Department of Translational Research on New Technologies in Medicine and Surgery, University of Pisa, Pisa, Italy; 26 Neuroradiology Unit, Azienda Ospedaliero-Universitaria Pisana, Pisa, Italy; 27 Australian Institute for Suicide Research and Prevention, Griffith University, Nathan, QLD Australia; 28 Slovene Centre for Suicide Research, University of Primorska, Koper, Slovenia; 29 De Leo Fund, Padova, Italy; 30 Section of Neurology, Department of Medicine and Surgery, University of Perugia, Perugia, Italy; 31 Unit of Medical Genetics, IRCCS Ospedale Policlinico San Martino, Genoa, Italy; 32 Department of Neuroscience, Rehabilitation, Ophthalmology, Genetics and Maternal and Child Health (DINOGMI), University of Genova, Genoa, Italy; 33 Immunology and Allergology Laboratory Unit, Ospedale San Giovanni di Dio, Azienda USL Toscana Centro, Florence, Italy; 34 Functional and Molecular Neuroimaging Unit, IRCCS Istituto delle Scienze Neurologiche di Bologna, Bologna, Italy; 35 Department of Biomedical and Neuromotor Sciences (DIBINEM), University of Bologna, Bologna, Italy; 36 Center for Neurodegenerative Diseases and the Aging Brain, University of Bari “Aldo Moro”, Tricase, Lecce, Italy; 37 Department of Translational Biomedicine and Neuroscience (DiBraiN), University of Bari “Aldo Moro”, Bari, Italy; 38 Department of Pharmacological and Biomolecular Sciences “Rodolfo Paoletti”, University of Milan, Milan, Italy; 39 Memory Clinic, Fondazione Policlinico Universitario “A. Gemelli” IRCCS, Rome, Italy; 40 Department of Neuroscience, Catholic University of the Sacred Heart, Rome, Italy; 41 Federazione Italiana Medici di Medicina Generale (FIMMG), Rome, Italy; 42 Division of Gerontology and Geriatrics, Department of Medicine and Surgery, University of Perugia, Perugia, Italy; 43 Division of Clinical Geriatrics, Department of Neurobiology, Care Sciences and Society, Karolinska Institutet, Stockholm, Sweden; 44 Division of Geriatric and Intensive Care Medicine, University of Florence & Azienda Ospedaliero-Universitaria Careggi, Florence, Italy; 45 Neurology Unit, Department of Clinical and Experimental Sciences, University of Brescia, Brescia, Italy; 46 Neurology Unit, Department of Continuity of Care and Frailty, ASST Spedali Civili di Brescia, Brescia, Italy; 47 Neurobiorepository and Advanced Biomarkers Laboratory, University of Brescia & ASST Spedali Civili di Brescia, Brescia, Italy; 48 Brain Health Center, University of Brescia, Brescia, Italy; 49 Unit of Geriatrics, Medical Department, San Giovanni-Addolorata Hospital, Rome, Italy; 50 Neurology and Stroke Unit, Luigi Sacco University Hospital, Milan, Italy; 51 Department of Biomedical and Clinical Sciences, Neuroscience Research Center, University of Milan, Milan, Italy; 52 Department of Neurorehabilitation Sciences, Casa di Cura Igea, Milan, Italy; 53 Department of Neuroscience, Psychology, Drug Research and Child Health (NEUROFARBA), University of Florence, Florence, Italy; 54 IRCCS Fondazione Don Carlo Gnocchi, Florence, Italy; 55 Fondazione Villa Serena per la Ricerca, Città Sant’Angelo, Pescara, Italy; 56 Department of Advanced Medical and Surgical Sciences, University of Campania “Luigi Vanvitelli”, Naples, Italy

**Keywords:** Alzheimer’s disease, Disease-modifying therapies, Anti-amyloid monoclonal antibodies, Biomarkers, Clinical implementation

## Abstract

This joint Position Paper, developed by the Italian Expert Panel on Alzheimer convened by the Italian Society of Neurology with participation from multiple scientific societies, outlines strategic guidelines for reorganizing the patient journey in the era of anti-amyloid monoclonal antibodies for Alzheimer’s disease. Emphasizing a multidisciplinary and integrated approach, the document recommends a patient journey that begins with early identification of cognitive impairment by General Practitioners, continues with specialized assessments at Memory and Dementia Centres, and leads, in carefully selected cases, to initiation of anti-amyloid monoclonal antibody therapy. It advocates the rational use of diagnostic tools, including plasma and cerebrospinal fluid biomarkers, advanced neuroimaging (MRI and PET), and genetic profiling (*ApoE* genotyping), not only to identify eligible patients but also to stratify those requiring alternative care strategies. The paper further defines minimum requirements for the accreditation of prescribing and infusion centres, highlighting the clinical competencies, structural resources, and inter-professional communication protocols necessary to ensure safety and appropriateness. Recognizing both the therapeutic potential and the organizational challenges associated with anti-amyloid monoclonal antibodies, the document aims to guide healthcare policymakers, institutions, and practitioners toward a coordinated reorganization of the diagnostic-therapeutic pathway, ensuring the safe and effective use of these treatments and ultimately improving outcomes and quality of care for individuals with Alzheimer’s disease.

## Introduction and aims

This joint Position Paper originates from the meeting of the “Expert Panel on Alzheimer (EPA)” held in Florence on January 24, 2025, organized by the Italian Society of Neurology (SIN), with representatives from numerous scientific societies, including the Academy of Geriatrics (AG), the Italian Association of Nuclear Medicine, Molecular Imaging and Therapy (AIMN), the Italian Association of Neuroradiology (AINR), the Italian Psychogeriatrics Association (AIP), the Italian Federation of General Practitioners (FIMMG), the IRCCS Neurosciences and Neurorehabilitation Network (RIN), the Italian Society of Clinical Biochemistry and Molecular Biology - Laboratory Medicine (SIBioC), the Italian Society of Pharmacology (SIF), the Italian Society of Gerontology and Geriatrics (SIGG), the Italian Society of Hospital and Territorial Gerontology (SIGOT), the Italian Society of Human Genetics (SIGU), the Italian Society of General Practitioners and Primary Care Physicians (SIMG), the autonomous association affiliated with SIN for the dementias (SINdem), the Italian Society of Neuropsychology (SINP), the Italian Society for Neuroscience (SINS), the Italian Society of Clinical Pathology and Laboratory Medicine (SIPMeL), and the Society of Hospital Neurological Sciences (SNO). Importantly, the Italian Alzheimer’s Disease patients’ Association (AIMA) was also involved, ensuring that the perspectives of patients and caregivers were included alongside those of clinicians and researchers.

The shared objective is to synthesize the main reflections that emerged and to formulate recommendations integrated with the latest evidence from the literature and clinical practice, in light of the approval of new anti-amyloid monoclonal antibodies for mild Alzheimer’s disease.

This document aims to recommend key steps along the entire patient journey, from the first contact with General Practitioners (GPs) to the possible eligibility for anti-amyloid monoclonal antibodies. Although only a minority of patients will ultimately receive such treatment, all individuals with suspected cognitive decline should benefit from structured pathways for diagnosis, prevention, and care. The paper therefore sets out recommendations for the diagnostic-therapeutic process and the roles of different professionals, provides guidance on the appropriate use of biomarkers and imaging, and defines the minimum requirements for prescribing and infusion centres, while also addressing organizational and clinical challenges. The added value of this Position Paper lies in two key contributions: updating Italian guidelines that no longer reflect current eligibility criteria for anti-amyloid monoclonal antibodies, and adapting international standards to the specific organizational framework of the Italian healthcare system. In this way, it not only bridges the gap between global recommendations and national practice but also provides concrete proposals for their sustainable implementation in Italy.

## Methods of consensus development

The panel reviewed the most recent evidence and international guidelines on dementia diagnosis and treatment, integrating them with the specific organizational needs of the Italian healthcare system. Consensus was achieved through open discussion, iterative revisions, and collective review of draft versions. Each participating Society contributed its expertise, ensuring a multidisciplinary perspective. When evidence was insufficient, recommendations were based on shared expert opinion. A writing group synthesized contributions, and the final text was formally approved by all members. The resulting document represents a shared, pragmatic framework intended to complement existing guidelines with actionable, context-specific recommendations.

## Current scenario and need for reorganization

In the field of Alzheimer’s disease, recent years have seen the emergence of anti-amyloid monoclonal antibodies, which have demonstrated efficacy in reducing cerebral amyloid burden and, in selected patients, slowing cognitive decline [1, 2]. Nevertheless, the translation of these therapies into real-world clinical practice requires a precise redefinition of diagnostic and care pathways, the establishment of standardized monitoring protocols, and robust organizational frameworks to ensure equity of access and patient safety [1–3].

Several countries have already developed appropriate use recommendations (AURs) to support the introduction of anti-amyloid monoclonal antibodies [2–7]. These documents, informed by clinical trial data, additional analyses, case reports, and expert feedback, converge on the need for strict eligibility criteria, biomarker confirmation, systematic MRI monitoring, and structured risk management protocols. They also emphasize the importance of multidisciplinary evaluation, the implementation of national or regional registries, and shared decision-making, including the recognition that anti-amyloid therapy represents a viable option only in a minority of cases. Taken together, these experiences show that monoclonal antibody therapies can only be implemented safely and effectively when embedded in reorganized care models with clear diagnostic, monitoring, and governance structures.

The Italian context, however, presents specific challenges. The current national dementia guidelines, published before the approval by EMA of anti-amyloid monoclonal antibodies, recommend biomarker-supported diagnosis only in individuals with dementia and provide a strong negative recommendation for their use in mild cognitive impairment (MCI) [8]. This stance is now outdated and evidently in direct contrast with international recommendations, which recognize biomarker testing as essential in MCI due to Alzheimer’s disease [9–14], precisely the population targeted by these new treatments.

Furthermore, the Italian healthcare system is undergoing reorganization under the Ministerial Decree 77/2022 (DM77), which defines new standards for community-based care. DM77 establishes “community homes” (“case della comunità”) as multidisciplinary hubs for chronic disease management, and strengthens the integration between primary care and specialist services [15]. Aligning the patient journey with DM77 principles is therefore critical to ensure that only those most likely to benefit reach highly specialized pathways for anti-amyloid therapy, while avoiding inappropriate overloading of centres for cognitive disturbances and dementias (CDCDs). In line with the recent SINdem Delphi Consensus, which highlighted the necessity of regional diagnostic, therapeutic and care pathways (PDTAs) consistent with the Italian national dementia plan [16, 17], our recommendations further emphasize the need for CDCD network reorganization and implementation of standardized care pathways, while expanding their scope to incorporate the safety requirements of disease-modifying therapies

In this evolving scenario, Italy urgently needs to define updated and nationally coordinated protocols that integrate biomarker-based diagnosis, risk stratification, MRI surveillance, and structured organizational models consistent with this evolving scenario. Without shared rules and standardized procedures, there is a risk of both diagnostic-therapeutic inappropriateness and inequity in access, jeopardizing the potential benefits of these innovative but resource-intensive therapies.

## Early detection and stratification of cognitive decline in primary care

The EPA’s recommendations for the “patient journey,” defined as the pathway from the GP to the dementia specialist, emphasize the importance of an integrated and timely approach for early identification of cognitive decline and its possible causes. GPs usually represent the first point of contact for patients and caregivers seeking advice regarding memory or cognitive complaints, and they play a pivotal role as gatekeepers to the healthcare system and coordinators of long-term care. According to the Italian national dementia guidelines [8], the GP is responsible for the early recognition of cognitive and behavioural changes, the collection of clinical and family history, and the administration of brief cognitive screening instruments, together with the prescription of first-line laboratory tests and basic neuroimaging to exclude reversible causes of impairment. In the presence of suspected dementia or rapidly progressive symptoms, the GP ensures timely referral to specialized services such as neurologists or CDCDs, thereby facilitating access to diagnostic confirmation and tailored treatment plans. Following diagnosis, the GP remains central to monitoring the clinical trajectory, managing comorbidities, supporting caregivers, and integrating hospital-based and community resources to guarantee continuity of care. Moreover, given the high prevalence of multimorbidity in this population, the GP oversees the management of concomitant chronic conditions and provides ongoing support to caregivers, addressing their psychological, social, and organizational burden. In this framework, the GP should systematically assess both frailty and cognition, preferably using standardized tools, to decide whether referral is more appropriate to a general specialist (neurologist, geriatrician, or psychiatrist) or directly to a CDCD.

Frailty evaluation is essential, since chronological age alone is an inadequate and often unreliable indicator of resilience [18, 19]. In individuals over 60 years of age, the primary care frailty index (PC-FI), developed using data from Italian primary care patients and validated in the Swedish national study on aging and care in Kungsholmen against 1-, 3-, and 5-year mortality, provides a validated tool for differentiating between patients with severe frailty and those with mild or no frailty [20]. The PC-FI can be integrated into the electronic platforms routinely used in primary care to allow systematic and reproducible frailty assessment, and since such integration is already available in some systems, the EPA calls for its adoption across all platforms to ensure equity and standardization nationwide [20, 21].

In parallel, cognitive screening should be performed with the general practitioner assessment of cognition (GPCog) [22, 23], complemented when appropriate by instruments such as the patient health questionnaire-9 (PHQ-9) to assess depressive symptoms or mild behavioural alterations [24–26]. The systematic use of these tools enables the early detection of alterations that, if neglected, may be erroneously attributed to normal aging or mere “benign forgetfulness,” when they may in fact represent the early signs of a neurodegenerative process.

If severe frailty is identified, the GP should preferably refer the patient to the community homes instead of the CDCD, as foreseen by the DM77. In these settings, a territorial multidisciplinary team, comprising neurologists, geriatricians, psychiatrists, internists, and social workers, carries out a comprehensive assessment that includes cognition but situates it within a broader multidimensional framework. In the context of advanced frailty and clinical complexity, pursuing an etiological diagnosis through high-level biomarker or imaging technologies may have limited clinical utility, since the disease trajectory is likely to evolve independently of β-amyloid reduction and therapeutic impact on prognosis is minimal [27]. For these patients, cognitive impairment represents only one aspect of a global picture dominated by multimorbidity and loss of autonomy. The aim is to reach a syndromic diagnosis of cognitive impairment or dementia while simultaneously evaluating comorbidities, functional status, behavioural and psychological symptoms, polypharmacy, and social needs. When clinically indicated, targeted investigations such as laboratory tests and basic neuroimaging may still be useful to exclude reversible causes (e.g., metabolic, endocrine, or nutritional disorders), but the therapeutic strategy is represented by a multidimensional intervention rather than cognitive-oriented treatments.

If, on the other hand, frailty is not severe and cognitive screening reveals impairment, the GP should proceed with the exclusion of potentially reversible causes by requesting basic blood tests and initial neuroimaging. A brain MRI without contrast is the preferred option, as it allows not only the exclusion of focal lesions, cerebrovascular disease, or reversible causes of dementia-like symptoms such as normal pressure hydrocephalus or chronic subdural haematoma but also provides essential information on cerebrovascular burden. Importantly, this baseline MRI should include standardized reporting with a minimum dataset (*e.g.,* Fazekas score, medial temporal lobe atrophy, microbleed count), enabling an initial assessment of potential eligibility for disease-modifying therapies. When MRI is not feasible, a CT scan may be used to rule out major structural or reversible causes, but in such cases further imaging will be required if the patient is considered for anti-amyloid therapy [10]. In the presence of distressing mood disturbances or anxiety symptoms, a psychological assessment should also be considered. When indicated, treatment with second-generation antidepressants may be initiated, since depressive disorders can mimic or worsen cognitive impairment [28, 29], and pharmacological treatment has been shown to improve associated cognitive symptoms. [30].

If no reversible conditions or alternative pathologies are identified, and cognitive decline of possible neurodegenerative or vascular origin is suspected, the patient should then be referred to a CDCD for confirmatory diagnostic work-up and definition of an appropriate therapeutic strategy.

This pathway ensures that each patient with suspected cognitive impairment is appropriately evaluated and directed to the most suitable level of care. By introducing frailty assessment as the first decision point, resources are allocated in a rational way: patients with advanced frailty or complex multimorbidity are referred to the community homes, where multidisciplinary management addresses their global needs, while patients with preserved resilience but suspected neurodegenerative diseases are referred to CDCDs for specialist diagnostic confirmation.

This stratification serves multiple purposes. First, it avoids overburdening specialist centres with patients unlikely to benefit from advanced diagnostic or therapeutic procedures; second, it guarantees timely identification and work-up of individuals in the earliest phases of disease, who are those most likely to benefit from disease-specific interventions such as anti-amyloid monoclonal antibodies; third it is intended to provide adequate responses to subjects with complex needs beyond cognitive impairment. In this way, the patient journey integrates efficiency with equity, ensuring that each person receives care aligned with their clinical condition, frailty status, and therapeutic prospects.

## Specialist evaluation in the CDCD

The EPA recommends that, to ensure timely and effective intervention, every person with suspected cognitive decline should access a sensitive diagnostic pathway, regardless of whether symptoms are already objectifiable (MCI or mild dementia) or only self-reported (subjective cognitive decline).

At the level of CDCDs, the initial assessment should include traditional screening tests, such as the mini mental state examination (MMSE), Montreal cognitive assessment (MoCA), or Boston cognitive assessment (BoCA), alongside neuropsychological tools capable of detecting even minimal alterations. The EPA recommends the progressive adoption of more advanced and sensitive tools (*e.g.*, “memory binding” tests or semantic-phonemic fluency discrepancy tests) [31, 32]. In addition, recently standardized instruments such as the I-UDSNB [33] should be considered, as they offer greater accuracy in discriminating individuals in the pre-MCI stage or those with subjective symptoms likely attributable to neurodegeneration.

The use of such instruments addresses the need to move beyond old tests based on outdated normative data and to identify cases that, under classical statistical neuropsychological methodology, might result in false negatives (i.e., erroneously deemed cognitively unimpaired). As neuropsychological assessment is a clinical act, each centre may select the most appropriate in-depth tests to identify ambiguous or atypically evolving cases.

When considering treatment with anti-amyloid monoclonal antibodies, however, the evaluation must be more stringent. Eligibility should be confirmed using instruments and thresholds aligned with those employed in pivotal trials, to ensure both safety and comparability with trial populations. In this context, a MMSE score of at least 20/30, which is roughly equivalent to a MoCA score of 13/30 [34], is required, together with a global clinical dementia rating (CDR) score of 0.5 or 1 [35, 36]. In specific cases, such as low educational attainment, limited language proficiency, or atypical clinical presentations of Alzheimer’s disease (*e.g.*, logopenic variant primary progressive aphasia) [37, 38], the CDR score may outweigh lower MoCA or MMSE values. To determine this global CDR score in clinical practice, each item may be scored using a simplified score based on the clinician’s observation and judgment, the neuropsychological assessment, the information from the patient and her or his caregiver and/or an instrumental activities of daily living (IADL) scale [39–41].

Together with neuropsychological assessment, CDCDs should provide a psychological and behavioural assessment, with multiple aims: (1) the identification of psychiatric conditions and psychoactive drugs, potentially associated with cognitive impairment [42], which might have been overlooked in primary care assessment; (2) the identification of a mild behavioural impairment, which may support the diagnosis of a neurodegenerative disease [43]; (3) the assessment of patient and caregiver preferences regarding available treatments, which is crucial for care planning in older multimorbid subjects [44], especially in the perspective of disease modifying treatments.

Contrary to current national guidelines, the EPA recommends that a biological diagnosis should be offered to patients with mild cognitive impairment or mild dementia (see next sections). A growing challenge, however, is subjective cognitive decline (SCD), which already represents a frequent reason for referral to memory clinics and may soon be accompanied by results from consumer-purchased blood-based biomarker tests. Such findings, if available, should never be interpreted in isolation but must be re-evaluated in specialist centres within a multidisciplinary framework that integrates clinical, neuropsychological, and imaging data. At present, the EPA does not advocate the routine use of biological diagnostics in patients with isolated SCD, given their uncertain specificity and the absence of evidence supporting treatment at this stage [45–47]. Exceptions may exist in selected cases where objective evidence of decline from baseline is documented through longitudinal cognitive testing or detailed neurobehavioral assessments. This is particularly relevant in individuals with high cognitive reserve, where test performances may still fall within normal ranges despite an ongoing pathological process. In such circumstances, and only when a measurable progression is detected, the cautious use of biomarkers may be considered appropriate, especially in view of potential targeted therapeutic interventions that could meaningfully alter the clinical trajectory.

Finally, the EPA recommends that individuals with mild cognitive impairment but without clear evidence of an underlying neurodegenerative disease or biological alteration, as determined through biomarker evaluation, as well as those with SCD, should be directed toward “prevention” programs or multidomain interventions, accompanied by scheduled follow-up evaluations. These include optimal management of cardiometabolic risk factors, promotion of healthy lifestyles, and psychological support [48, 49]. This approach ensures appropriate follow-up, avoids premature labelling or overtreatment, and provides a clear pathway for re-entry into the diagnostic framework if and when progression emerges. If, over time, clinical or biological indicators of a neurodegenerative process appear, further diagnostic testing and potential disease-modifying treatment may then be warranted.

Given that frailty is a dynamic and potentially progressive condition, patients initially considered eligible for anti-amyloid therapy may experience functional or cognitive deterioration before or during treatment. For this reason, frailty assessment at the CDCD should extend beyond diagnostic confirmation to include a proactive evaluation of vulnerable health domains (*e.g.,* physical, nutritional, psychosocial), with the aim of enabling personalized, multidisciplinary interventions that support resilience, reduce the risk of clinical worsening, and ensure continuity of care and sustained therapeutic appropriateness.

Finally, it should be noted that the present document does not address in detail the necessary pathways of post-diagnostic care and monitoring for individuals with overt dementia, including the management of behavioural and psychological symptoms, the cognitive effects of drug treatment, caregiver support and counselling, psychosocial interventions, and advance care planning. These aspects remain crucial and should be developed through complementary guidelines and integrated into a broader continuum of dementia care.

## The role of MRI: standardized criteria and protocols

The EPA assigns a central role to MRI both in the initial diagnostic assessment of patients with suspected early Alzheimer’s disease and in the longitudinal monitoring of those treated with anti-amyloid monoclonal antibodies. MRI should represent an early step of the pathway and ideally already be available when the patient reaches the specialist level.

At the diagnostic stage, MRI plays a fundamental role with several complementary functions. It helps rule out alternative non-neurodegenerative causes of cognitive decline, while also characterizing cerebrovascular comorbidities by assessing the severity of small vessel disease, the number of microbleeds, and the presence of superficial siderosis [50–52]. These findings not only inform the etiological diagnosis but also provide a baseline for determining eligibility for treatment and for monitoring safety during follow-up. Clinical trial evidence has shown that the early identification of such features is essential, as they help to detect patients at increased risk of amyloid-related imaging abnormalities (ARIA), including haemorrhages and vasogenic oedema, which require careful surveillance throughout therapy [2, 3, 35, 36, 53, 54].

If MRI cannot be performed, treatment with current anti-amyloid monoclonal antibodies is effectively ruled out. Patients must be able to safely undergo MRI at 1.5 or 3 Tesla and should have no contraindications. The first MRI should therefore be performed as soon as possible once a therapeutic indication is considered, and in any case within 6 months before therapy initiation. New or unusual neurological symptoms within these 6 months should prompt a new MRI.

Regarding imaging exclusion criteria, the EPA is in line with other published AURs [2, 3, 5, 7], which specify that patients should be excluded if baseline MRI shows amyloid-related imaging abnormalities of oedema/effusion, more than four cerebral microhaemorrhages, any cortical superficial siderosis, any intracerebral haemorrhage greater than 1 cm, severe white matter disease (Fazekas 3) [55], probable CAA (Boston 2.0 criteria) [56, 57], criteria for CAA-related inflammation [58], territorial infarcts larger than 1 cm, more than two lacunar infarcts, cerebral contusion, encephalomalacia, brain aneurysms or other vascular malformations, central nervous system infection, or brain tumours except for small meningiomas or arachnoid cysts (see proposed inclusion/exclusion criteria in Table 1).

**Table 1:** Proposed inclusion and exclusion criteria of the Italian appropriate use recommendations for lecanemab and donanemab (adapted from [2, 3, 5, 7])

Inclusion criteria	
Clinical diagnosis of MCI or mild AD dementia [59, 60]	
Positive CSF (A+/T+) or amyloid PET (based on visual read) indicative of AD	
No exclusion based only on chronological age; multidisciplinary discussion for extremes <50 or >90 years	
MMSE 20–30, MoCA 13–30. CDR global score of 0.5 or 1. Clinician judgement in individuals with low educational attainment, limited language proficiency, or atypical clinical presentations	
Amnestic (typical) AD phenotype, or other non-amnestic common AD phenotypes (logopenic variant primary progressive aphasia or posterior cortical atrophy), multidisciplinary discussion in situations where the link between Alzheimer’s pathophysiology and the clinical phenotype is less straightforward (corticobasal syndrome, behavioural and dysexecutive variants, or the non-logopenic primary progressive aphasias)	
Clinical *ApoE* genotyping prior to initiating treatment	
Patients may be on cognitive enhancing agents (donepezil, rivastigmina, galantamine, or memantine) for AD; patients may not be on other anti-amyloid monoclonal antibodies	
Have a care partner or family member who can ensure that the patient has the support needed to be treated with lecanemab or donanemab	
Patients and their care partners should understand the requirements for lecanemab or donanemab therapy and the potential benefit and potential harm of treatment	
Baseline brain MRI performed within 6 months prior to treatment initiation, including standardized sequences (2D/3D FLAIR, T2 GRE±SWI, DWI, 3D T1, T2 FSE), to establish eligibility and ARIA risk profile	
Exclusion criteria	
Any medical, neurologic, or psychiatric condition that may be contributing to the cognitive impairment or any non-AD MCI or dementia	
Recent history (within 12 months) of stroke or transient ischemic attacks or any history of seizures	
Psychiatric disorder that interferes with comprehension of the requirements, potential benefit, and potential harms of treatment and are considered by the physician to render the patient unable to comply with management requirements; patients for whom disclosure of a positive biomarker may trigger suicidal ideation	
Any history of systemic immunologic disease (e.g., lupus erythematosus, rheumatoid arthritis, Crohn’s disease) or systemic treatment with immunosuppressants, immunoglobulins, or monoclonal antibodies or their derivatives	
Unstable medical conditions that could increase the risk of adverse events or interfere with treatment and monitoring; a condition of severe frailty	
Contraindications for MRI, including claustrophobia or the presence of contraindicated metal (ferromagnetic) implants/cardiac pacemaker	
Abnormality on baseline MRI suggesting a non-AD cause for progressive cognitive impairment	
More than 4 microhaemorrhages (defined as <10 mm at greatest diameter); a single macrohaemorrhage (>10 mm at greatest diameter); an area of superficial siderosis; evidence of vasogenic oedema; more than 2 lacunar infarcts or stroke involving a major vascular territory; severe subcortical hyperintensities consistent with a Fazekas score of 3; evidence of amyloid beta-related angiitis (ABRA); probable cerebral amyloid angiopathy (CAA) Boston 2.0 criteria; cerebral amyloid angiopathy-related inflammation (CAA-ri); or other major intracranial pathology that may cause cognitive impairment; multidisciplinary discussion for unruptured intracranial vascular malformations	
Patients with a bleeding disorder that is not under adequate control (including a platelet count <50,000/uL or international normalized ratio (INR) >1.5 for participants who are not on anticoagulants	
Patients on anticoagulants (coumadin, dabigatran, edoxaban, rivaroxaban, apixaban, betrixaban or heparin) should not receive lecanemab or donanemab; tPA should not be administered to individuals on lecanemab or donanemab	
* ApoE ε4* homozygotes are not eligible for lecanemab or donanemab treatment	

*AD* Alzheimer’s disease, *MCI* mild cognitive impairment, *CSF* cerebrospinal fluid, *A+/T+* amyloid-positive/tau-positive, *PET* positron emission tomography, *MMSE* Mini-Mental State Examination, *MoCA* Montreal Cognitive Assessment, *CDR* Clinical Dementia Rating, *ApoE* apolipoprotein E, *MRI* magnetic resonance imaging, *ARIA* amyloid-related imaging abnormalities, *FLAIR* fluid-attenuated inversion recovery, *GRE* gradient-recalled echo, *SWI* susceptibility-weighted imaging, *DWI* diffusion-weighted imaging, *FSE* fast spin echo, *ABRA* amyloid-β–related angiitis, *CAA* cerebral amyloid angiopathy, *CAA-ri* CAA-related inflammation, *INR* international normalized ratio, *tPA* tissue plasminogen activator

Regarding acquisition protocols, the EPA recommends that the baseline MRI study follows the standards endorsed by American and European neuroradiology societies [50]. This includes 2D or 3D T2 FLAIR, T2 GRE±SWI, DWI, 3D T1, and T2 FSE. Standardized axial T2* GRE with appropriate TE should be performed in all patients (TW=15–20 ms at 3 T and 25–35 ms at 1.5T). While SWI sequences are more sensitive to haemosiderin deposits, GRE sequences were preferred in clinical trials because of lower variability across scanners and broader availability. The inclusion of SWI may still be helpful where feasible [61], but this decision should be shared with the treating physician as it may result in more restrictive eligibility thresholds. In addition, a standardized reporting dataset should be completed for all baseline MRIs, even when performed outside reference centres. At minimum, this should include the Fazekas score for white matter hyperintensities, a medial temporal lobe atrophy rating, and the number of cerebral microbleeds, to ensure consistent and reproducible evaluation of treatment eligibility and safety across centres.

Once anti-amlyoid monoclonal antibody therapy is initiated, MRI becomes essential for safety monitoring. In asymptomatic patients, follow-up protocols may be simplified to include 2D or 3D T2 FLAIR, GRE±SWI, DWI sequences. In contrast, when ARIA is suspected, the MRI protocol should be extended with additional sequences tailored to the clinical scenario to allow full differential diagnosis (infarct, tumour/metastases, infections) [50].

The effective use of MRI in this context requires strong communication channels between clinicians, neuroradiologists, and patients. Specialists interpreting MRI scans should systematically report the severity and extent of any ARIA, quantifying microbleeds and characterizing oedema as per drug data sheets, following the guidelines suggested by American and European neuroradiology societies, so that neurologists and geriatricians can decide whether to suspend, continue, or adjust the dosage of the drug [50]. Simultaneously, patients and caregivers must be informed about how to act in case of symptoms that may require urgent MRI, and the importance of carrying up-to-date documentation (or a digital copy) to facilitate comparisons with previous scans.

To support such a system, the EPA recommends defining minimum requirements (detailed below) for centres managing these patients, both in terms of technical equipment and staff expertise. Targeted training courses should be promoted to educate neuroradiologists and general radiologists on recognizing ARIA. Moreover, national standardization of protocols, the possibility of teleconsultation in a hub-and-spoke model, and “certification” of centres to ensure quality could all promote uniform and safe management of the growing number of patients eligible for anti-amyloid therapy.

## Stepwise biomarker strategy: from biological diagnosis to treatment eligibility

The EPA emphasizes that the choice of biomarkers and their interpretation must be guided by the clinical context, distinguishing between patients who require only a biological diagnosis and those who are being evaluated for treatment eligibility.

In patients with mild cognitive impairment or mild dementia, when the sole objective is to establish a biological diagnosis, a positive result on a plasma biomarker assay using a validated double-threshold approach may be considered adequate. Such assays, particularly those based on p-tau_217_ or the p-tau_217_/amyloid-β_42_ ratio, have demonstrated accuracy exceeding 95% against CSF and amyloid PET [62–67]. Their adoption is further supported by recent FDA authorization of plasma p-tau_217_/amyloid-β_42_ for diagnostic use [68, 69]. In this context the EPA recommends applying a double-threshold framework: values above the high threshold, set to achieve at least 97.5% specificity, classify patients as positive, yielding a positive predictive value ≥99% when the pre-test probability is high (as in typical amnestic Alzheimer’s or other common phenotypes) [7]. Values below the lower threshold classify as negative, whereas intermediate results (“grey zone”) require confirmatory testing with either CSF or PET. This stepwise strategy both minimizes the risk of misclassification and ensures sustainable use of healthcare resources [67, 70–73]. This is in line with recent guidelines by the Alzheimer’s Association on the use of blood-based biomarkers within specialized care settings [74].

By contrast, when patients are considered potential candidates for anti-amyloid monoclonal antibody therapy, confirmation with CSF or PET is deemed mandatory at the present time. The EPA recommends CSF analysis as the first-line confirmatory test. This should demonstrate a significant reduction in amyloid-β_42_ (ideally interpreted through ratios such as amyloid-β_42/40_) together with an abnormal increase in p-tau_181_ [75]. In cases where CSF findings fall into a borderline range (for example, when the amyloid ratio lies within 10% of the diagnostic threshold) yet the patient presents with a T+ status and a common Alzheimer’s phenotype, amyloid PET should be performed to exclude false negatives [5, 76, 77].

At present, there is limited evidence to recommend treatment in patients with an A+T- profile. However, lower levels of tau pathology have been associated with better clinical outcomes [36], suggesting that borderline or grey-zone p-tau results may still be acceptable for treatment initiation in carefully selected patients with a typical phenotype [78].

Amyloid PET plays a complementary but distinct role within this framework. From a diagnostic standpoint, it is primarily indicated when CSF is contraindicated, refused, or yields inconclusive results [10], as it provides direct evidence of amyloid deposition with strong concordance to neuropathological findings and high sensitivity and specificity [79]. In this setting, the presence of diffuse cortical amyloid deposition (A+/T?) on PET, combined with a common Alzheimer’s phenotype, may be considered sufficient to establish eligibility for therapy [80] (see proposed inclusion/exclusion criteria in Table 1).

Caution is warranted when interpreting results in older patients (>80 years), since reduced CSF amyloid-β_42_ and amyloid PET positivity may occur even in the absence of cognitive impairment or in conditions other than Alzheimer’s disease [81, 82]. The EPA emphasizes, however, that age alone should not be used to exclude patients from CSF testing. Instead, decisions should be guided by validated frailty indices, which represent reliable proxies of biological resilience [83–86].

Dual-phase amyloid PET imaging can further enhance diagnostic yield by combining information on amyloid deposition (late phase) with cerebral perfusion (early phase), which provides functional data partly comparable to FDG-PET in identifying regional patterns of neurodegeneration and improving differential diagnosis among dementia phenotypes [87–90]. FDG-PET nonetheless remains more sensitive for detecting early synaptic dysfunction and remains a valuable complementary tool when available [79, 91, 92].

Beyond diagnosis, PET assumes a unique role in therapy monitoring, as it remains the only available method that reliably demonstrates amyloid clearance following treatment, making it the gold standard for evaluating biological response [35, 36, 93, 94]. This distinction carries practical implications: while lecanemab requires indefinite treatment continuation irrespective of amyloid clearance [35], in the case of donanemab, therapy could be suspended once PET confirms removal of amyloid to subthreshold levels [36].

Although desirable, a baseline amyloid PET scan prior to treatment initiation should not be considered mandatory, and its absence should not delay therapy in otherwise eligible patients, to avoid missing the optimal therapeutic window [95]. When access allows, however, a baseline PET followed by a repeat scan around 12 months, or at an interval tailored to the clinical context, can provide valuable information for guiding treatment continuation or discontinuation.

Another key issue raised by the EPA is the integration of PET with MRI. Structural imaging (MRI) helps detect co-pathologies such as amyloid angiopathy or vascular lesions that increase the risk of complications during anti-amyloid therapy, while PET provides complementary “functional” or “pathophysiological” information valuable for differential diagnosis and prognosis. Looking forward, hybrid imaging systems (PET/MRI) and high-sensitivity dedicated PET scanners (smaller and potentially more cost-effective) may improve access and reduce organizational burden [96].

The EPA recommends moving beyond a simple binary “positive/negative” interpretation by adopting quantitative measures such as the Centiloid scale, which harmonizes standardized uptake value ratios (SUVr) across tracers and imaging protocols [95, 97, 98]. This tracer-independent metric enables consistent comparison of amyloid burden between centres and across disease stages, enhancing both diagnosis and longitudinal monitoring.

Sustainability remains a key concern. Widespread PET availability is currently unfeasible; therefore, PET should be prioritized where it has a real impact on clinical decision-making, such as when lumbar puncture is contraindicated, in uncertain cases, or to discontinue therapies that have already achieved a biological endpoint. This rationale highlights the need for close collaboration among nuclear medicine physicians, neurologists, geriatricians, and radiologists to ensure shared protocols and judicious use of imaging.

Moreover, specific training, access to automated analysis software, and the validation of shared criteria (*e.g.*, thresholds for meaningful amyloid reduction) are all essential to convert the potential of PET into actual clinical benefit.

Beyond the biomarker framework, treatment decisions must also take into account the clinical phenotype, as not all presentations of Alzheimer’s disease are equally appropriate for anti-amyloid therapy. Based on current knowledge, anti-amyloid monoclonal antibodies should be offered primarily to patients with common Alzheimer’s phenotypes, which include the typical amnestic syndrome [99], the logopenic variant of primary progressive aphasia [100], and posterior cortical atrophy [101]. These phenotypes show amyloid and tau biomarker profiles comparable to typical Alzheimer’s disease, with strong concordance between CSF and amyloid PET [76, 99–107]. Moreover, the prevalence of *ApoE ε4* carriage, and thus ARIA risk, is lower in these atypical but biologically common phenotypes than in amnestic subjects included in pivotal trials [108, 109], suggesting an overall acceptable risk-benefit balance [110]. Treatment in these groups is therefore considered appropriate, although additional factors such as age, sex, tau pathology burden, mixed brain pathologies and psychiatric comorbidities [109, 111, 112] should always be carefully weighed in multidisciplinary discussions [5, 7].

By contrast, caution is warranted in less common phenotypes such as corticobasal syndrome [113], the behavioural and dysexecutive variants [104, 114], or the non-logopenic primary progressive aphasias [100]. In these conditions, Alzheimer’s pathology is often only a comorbidity [102, 115–118], making the therapeutic benefit of anti-amyloid immunotherapy limited while risks persist. Similar caution applies to patients with mixed pathologies (*e.g.*, Alzheimer’s plus Lewy body disease) or with other atypical presentations, where biomarker positivity is more likely to indicate secondary rather than primary Alzheimer’s pathology [112, 119]. To date, no clinical efficacy has been demonstrated in these groups, nor in asymptomatic biomarker-positive individuals. We therefore recommend that treatment indications be discussed in multidisciplinary settings such as memory boards, particularly in situations where the link between Alzheimer’s pathophysiology and the clinical phenotype is less straightforward.

Eligibility should not be determined by chronological age alone. In accordance with other AURs [2–5, 7], we emphasize that patients outside the age ranges of pivotal trials (50–90 years for lecanemab, 65–85 years for donanemab) should not be automatically excluded but rather discussed in multidisciplinary meetings. In these cases, careful consideration of comorbidities is required, and this should always include a structured assessment of multimorbidity and frailty. Trial exclusion criteria capture discrete risks but do not reflect the multidimensional vulnerability that characterizes complexity and frailty across physical, cognitive, and social domains. Conditions systematically included in the primary care frailty index, such as recent hospitalizations, multimorbidity, polypharmacy, or social disadvantage, may not formally exclude a patient if considered “stable,” yet are strong predictors of poor tolerance, complications, and discontinuation in real-world practice [120, 121]. Severely frail individuals should therefore be excluded even within the canonical age range, while robust patients who fall outside trial limits may still be considered if overall evaluation suggests sufficient resilience to tolerate therapy and monitoring.

## The role of ApoE genotyping

*Apolipoprotein E* (*ApoE*) genotyping has long been of interest, as the *ε4* allele represents the strongest genetic risk factor for sporadic Alzheimer’s disease and for cerebral amyloid angiopathy [122–124]. However, while its epidemiological impact is well established, *ApoE* genotyping lacks individual predictive value: carrying the *ε4* allele increases disease risk, but this risk varies depending on age, sex, other genetic variants, vascular risk factors, and lifestyle [49, 125]. Some evidence suggests that in very old individuals, especially those over 90, the impact of the *ε4* allele on Alzheimer’s risk and survival is significantly reduced compared to younger individuals [126].

As a result, *ApoE* genotyping for purely predictive purposes is discouraged in the absence of appropriate genetic counselling, in line with recommendations from major international scientific societies [127, 128].

With the advent of anti-amyloid therapies, *ApoE* status has become a central determinant of both eligibility and risk stratification. A robust body of evidence shows that *ApoE* ε4 carriers, especially homozygotes, have a substantially higher risk of ARIA, including symptomatic and recurrent forms, an increased risk of CAA-related inflammation and amyloid- β-related angiitis, as well as differences in therapeutic efficacy [35, 36, 53, 54]. For this reason, genotyping should be systematically performed in all patients considered for treatment with anti-amyloid monoclonal antibodies. At the present time *ApoE ε4* homozygotes should not receive these therapies, as the risk of harm outweighs potential benefit, in line with EMA guidance. However, recent studies suggest that modified titration schemes may reduce the incidence and severity of ARIA-E in both *ApoE ε4* homozygotes and heterozygotes. For heterozygous carriers, results should inform an individualized monitoring protocol and be discussed transparently with patients and caregivers as part of pre-treatment counselling.

Further, ongoing research, including studies on drugs targeting *ApoE* function, could broaden the potential applications of genotyping, particularly when integrated with assessments of biological versus chronological age [129, 130].

It is thus essential that *ApoE* genotyping be considered within a multidimensional framework, including biomarker data, MRI findings, detailed risk factor history, and, especially in older individuals, frailty evaluation. The EPA explicitly recommends that ApoE genotyping should be performed only after adequate patient preparation and with the availability of pre- and post-test genetic counselling, normally delivered by a geneticist, or, in cases when this is not feasible, by a dementia specialist with documented expertise in genetic result interpretation [127]. This approach ensures responsible result management, prevents misunderstandings or unfounded concerns, and reinforces the fact that *ApoE ε4* carriage does not necessarily imply progression to Alzheimer’s disease or increased risk of cerebral haemorrhage. When appropriately contextualized, this information can contribute to more rational and personalized use of new treatments.

## Summary of the patient journey

The EPA outlines a diagnostic-therapeutic pathway grounded in close collaboration between GPs and CDCDs. The initial step involves a GP-led evaluation that always includes frailty assessment and cognitive screening (*e.g.*, GPCog), together with basic laboratory tests to exclude reversible causes. If the patient’s frailty is not classified as severe and the clinical picture raises suspicion of a neurodegenerative condition, the GP rules out secondary causes of dementia and requests an initial neuroimaging study, preferably an MRI scan with standardized reporting, and, in the absence of reversible causes of cognitive decline or severe frailty, refers the patient to the CDCD.

At the CDCD, a multidisciplinary clinical assessment and detailed cognitive testing should be conducted. Plasma biomarkers, particularly assays such as p-tau_217_ or the p-tau_217_/amyloid-β_42_ ratio, may provide the first step toward establishing a biological diagnosis. When the goal is limited to confirming the presence or absence of Alzheimer’s pathology, a validated double-threshold approach can be applied: values above the upper threshold reliably support the diagnosis, while values below the lower threshold effectively exclude it. Only those results that fall into an intermediate “grey zone” require confirmation with CSF analysis or amyloid PET. By contrast, when treatment eligibility for anti-amyloid monoclonal antibodies is under consideration, plasma biomarkers alone are not sufficient at the present time. In these cases, CSF analysis should be regarded as the first-line confirmatory test, requiring both evidence of amyloid reduction and abnormal tau elevation. If the CSF profile lies close to diagnostic cut-offs or appears discordant with the clinical presentation, amyloid PET should then be performed to resolve diagnostic uncertainty, and it also serves as an alternative when lumbar puncture is not feasible.

Once a neuropathological process consistent with Alzheimer’s disease is confirmed, and the clinical presentation is compatible with common Alzheimer’s phenotypes, the patient enters the pre-therapeutic phase.

At this point, *ApoE* genotyping is recommended, as it defines the eligibility of patients for anti-amyloid monoclonal antibody therapy and assess the risk of vascular complications and ARIA. If not already performed earlier, it should be carried out before treatment begins.

If all investigations confirm the patient’s clinical and biological eligibility, anti-amyloid monoclonal antibody therapy can be initiated. The EPA recommends, where feasible, acquiring a baseline amyloid PET to more precisely document amyloid burden prior to therapy. However, delayed access to PET should not preclude treatment initiation, as this may jeopardize the optimal treatment window.

In cases where the patient is deemed ineligible for treatment, appropriate care should still be provided, including psychological counselling for the patient and family.

During therapy, regular MRI monitoring is recommended, according to each drug’s specific guidelines, to detect potential complications such as ARIA-E or ARIA-H. In a later phase, repeat amyloid PET may be needed to assess whether amyloid burden has decreased below a biologically relevant threshold, and to consider discontinuation of therapy for certain drugs. In the future, this reassessment may be guided by plasma biomarkers, provided their reliability for monitoring purposes is validated.

The overarching goal is to ensure rigorous diagnostics, timely treatment initiation, and targeted monitoring, thereby optimizing both resource use and patient safety.

## Minimum requirements for the accreditation of prescribing and infusion centres

The EPA emphasizes the need to clearly define the minimum requirements that a centre must meet to safely prescribe and administer disease-modifying drugs, given the clinical and organizational complexity these therapies entail. The standards that follow are supported, whenever possible, by the available literature, including international AURs, and, where evidence is still limited, by multidisciplinary expert consensus.

It is essential to distinguish between prescribing centres, which are responsible for patient selection and treatment indication, and infusion centres, which are responsible for the safe administration and monitoring of therapy.

Prescribing centres should have a clinical team with advanced diagnostic expertise, including specialists in the assessment and management of cognitive impairment, supported by neuropsychologists and trained nursing staff [16, 131]. These centres should be able to identify patients with mild cognitive impairment or mild dementia due to Alzheimer’s disease, perform structured neuropsychological evaluations, interpret biomarker data (CSF, PET, plasma assays), and manage the patient's biological, somatic and psychosocial complexity (*i.e.,* frailty) [2–5, 7, 15, 16, 132]. They must also ensure access to MRI with standardized protocols to establish cerebrovascular comorbidities and ARIA baseline risk [2–5, 7, 15, 16, 50, 51, 131–133]. Furthermore, prescribing centres must provide comprehensive informed consent procedures [134], incorporating genetic counselling when *ApoE* genotyping is performed [2, 3, 5, 7, 133], and clearly communicate both risks and expected benefits of treatment. Electronic medical records and contribution to national or regional registries are required to guarantee transparency, appropriateness, and harmonization across the healthcare system [5, 135] (see Table 2).

**Table 2:** Minimum requirements for the accreditation of prescribing centres for anti-amyloid monoclonal antibody treatment in Alzheimer’s disease

Requirement	Details
Advanced diagnostic expertise	Multidisciplinary team (neurologist and/or geriatrician and/or psychiatrist, neuropsychologist, dedicated nursing staff) capable of identifying early disease stages, performing neuropsychological assessments, and interpreting biological tests (CSF/plasma), with full competence of inclusion/exclusion criteria.
Capacity for biological profiling	Ability to perform or coordinate confirmatory testing (*e.g.*, lumbar puncture, amyloid PET). Collaboration with specialized laboratories (for CSF, plasma biomarkers, and genetic testing) or nuclear medicine centres (PET).
Frailty assessment	Use of validated tools to estimate clinical complexity (including frailty scales and comorbidity indices) to guide diagnostic and therapeutic decisions.
Neuroradiological support	Access to imaging (CT and MRI) performed using standardized protocols aimed to exclude possible mimickers, identifying neurodegenerative changes, and detecting microbleeds or superficial siderosis.
Integrated monitoring plan	Computerized system for clinical data collection and Alzheimer’s disease registry, including neuropsychological tests, laboratory data, and imaging for structured follow-up.
Counselling and informed consent	Ability to provide comprehensive information to patients and families, including genetic aspects (*ApoE*) and potential treatment complications, within a structured informed consent framework.

*ARIA* amyloid-related imaging abnormalities, *CSF* cerebrospinal fluid, *CT* computerized tomography, *MRI* magnetic resonance imaging, *PET* positron emission tomography

Infusion centres must guarantee a structural and organizational setting suitable for safe treatment delivery. This includes an infusion unit staffed by trained physicians and nurses experienced in intravenous therapies and infusion reaction management, as well as a hospital pharmacy capable of preparing monoclonal antibodies under controlled conditions [2–5, 7, 15, 16, 132]. Continuous access to MRI (1.5T or 3 T) must be available both for baseline evaluations and urgent scans, with radiologists proficient in the detection of ARIA and able to distinguish ARIA-E from ARIA-H [2–5, 7, 15, 16, 50, 51, 131–133]. Because ARIA may present with heterogeneous or non-specific symptoms, access to neurological expertise, particularly neurologists experienced in the management of seizures, status epilepticus, and cerebral oedema, is strongly recommended to support dementia specialists in diagnosis, follow-up, and treatment-related decisions [2–5, 7, 15, 16, 50, 51, 131–133]. Emergency and intensive care services (either within the hospital or through closely networked facilities) must be available for severe complications, with EEG services accessible to support the management of seizures and status epilepticus [2–5, 7, 15, 16, 50, 51, 131–133]. Close collaboration between treating physicians, neuroradiologists, and nuclear medicine specialists is essential, and standard operating procedures (SOPs) for both infusion-related adverse events and ARIA must be in place, signed by all involved relevant professionals and regularly updated (see Table 3) [2, 3, 7, 131].

**Table 3:** Minimum requirements for the accreditation of infusion centres for anti-amyloid monoclonal antibody treatment in Alzheimer’s disease

Requirement	Details
Appropriate hospital infrastructure	Availability of a dedicated infusion area, with medical and nursing staff trained in the administration of intravenous therapies and the management of potential infusion-related or neurological adverse events.
Emergency department and ward	Availability of an emergency department (within the hospital or closely networked) capable of promptly identifying and managing ARIA or other complications; inpatient wards (neurology or geriatrics) with dedicated beds for patient observation and monitoring.
Rapid access to MRI	Availability of magnetic resonance imaging (including 24/7 emergency access) for detection of ARIA or other acute lesions; need for neuroradiologists and radiologists with expertise in ARIA interpretation.
Clinical expertise in ARIA management	Clinicians experienced in managing cerebral oedema, subclinical haemorrhages, and ARIA, with capacity for emergency intervention (including continuous coordination with anaesthesiology/intensive care and neuroradiological consultation).
Intensive care unit	Access to an intensive care unit in case of severe complications.
Neurophysiology unit	Availability of electroencephalography (EEG) equipment for inpatients, to assess possible status epilepticus.
Efficient communication system	Continuous linkage with diagnostic specialists (e.g., nuclear medicine, neuroradiology); real-time information exchange for reassessment or treatment discontinuation, if needed.
Structured hospital pharmacy	Internal pharmacy service qualified to manage, store, and prepare disease-modifying drugs with appropriate safety protocols.
Registry and follow-up	Electronic platform for tracking infusion cycles, adverse event monitoring, and clinical progression of the patient.

*ARIA* amyloid-related imaging abnormalities, *EEG* electroencephalography, *MRI* magnetic resonance imaging

Although the number of patients eligible for treatment is expected to remain relatively small compared to the overall prevalence of dementia, the complexity of management requires a sustained hospital commitment, with infusions scheduled every two to four weeks and readiness to promptly address complications. Experience from other models, such as stroke networks, demonstrates the importance of integrating neurology, radiology, and intensive care expertise to ensure safety. A mothership model may represent the most sustainable approach, with advanced diagnostic and prescribing activities concentrated in reference centres and infusions delivered in accredited centres, provided all safety standards are met and effective communication with the prescribing centre is guaranteed.

Finally, each accredited centre must operate within a coordinated network and contribute to patient registries that monitor eligibility, outcomes, and adverse events. While future therapies with improved safety profiles may eventually allow for a relaxation of these requirements, the current generation of anti-amyloid monoclonal antibodies demands rigorous standards for prescribing and infusion centres to ensure safe, equitable, and sustainable implementation in clinical practice [136].

## Conclusions

The EPA considers the advent of anti-amyloid drugs a unique opportunity to modify the course of Alzheimer’s disease in its early stages, when cognitive decline is still mild and has minimal impact on autonomy in daily life. At the same time, this therapeutic innovation presents significant challenges, both in terms of precisely identifying eligible patients and managing the complex clinical and organizational aspects of monitoring and potential complications.

The development and adoption of shared diagnostic protocols and the upgrading of prescribing centres, including infusion facilities, are therefore fundamental prerequisites for ensuring accessibility, appropriateness, and safety.

The definition of a clear diagnostic pathway; the rational use of fluid biomarkers, imaging methods, and *ApoE* genotyping; and the establishment of centres with proven expertise and adequate infrastructure are all essential elements for fully realizing the potential of these new treatments.

In parallel, education and collaboration among multiple professional roles, including general practitioners, geriatricians, neurologists, neuroradiologists, nuclear medicine physicians, psychiatrists, pharmacologists, and psychologists, will be increasingly critical to ensure a multidisciplinary approach centred on the patient and their needs.

Recent studies indicate that, when applying criteria from randomized controlled trials or appropriate use recommendations for donanemab and lecanemab, only about 10% of patients with mild cognitive impairment or mild dementia in tertiary centres meet eligibility requirements [137, 138]. This finding aligns with previous real-world observations for aducanumab [139] and underscores the importance of developing patient journey models reflecting realistic treatment numbers, thereby promoting the appropriate use of these therapies in clinical practice.

The EPA emphasizes that, alongside clinical and organizational measures, clear public communication will be essential for the responsible implementation of anti-amyloid therapies. The growing media attention surrounding these drugs, combined with increasing engagement of grassroots associations and political debate, risks generating unrealistic expectations and inappropriate demand. To avoid overburdening specialist centres and ensure equitable access, public information campaigns should prioritize transparent communication that these therapies are restricted to selected patient subgroups, with biomarker-confirmed Alzheimer’s disease and within defined eligibility criteria.

Collaboration with patient associations is essential, as it helps align expectations with scientific evidence, fosters awareness of prevention strategies, and supports patients and families in navigating appropriate diagnostic and therapeutic pathways.

Policymakers should also address the critical shortage of GPs, whose numbers are progressively declining [140], by ensuring adequate workforce and providing them with sufficient resources and diagnostic prescribing capabilities. In parallel, CDCDs must be reinforced with additional multidisciplinary staff, including neurologists, geriatricians, psychiatrists, psychologists, and dedicated nursing personnel, to deliver accurate diagnostic work-ups and therapeutic planning. Although these professional figures are explicitly mandated by the Italian national dementia plan [16], they remain unavailable in many centres, generating inequities in patient access and care across regions [141].

The hope is that the guidance provided in this Position Paper will contribute to the balanced implementation of disease-modifying therapies, to the benefit of the entire community and in alignment with the evolving landscape of research and clinical practice.

## Abbreviations

ApoE: Apolipoprotein E
ARIA: Amyloid-related imaging abnormalities
BoCA: Boston cognitive assessment
CDCD: Centre for Cognitive Disorders and Dementia
CSF: Cerebrospinal fluid
CT: Computed tomography
DWI: Diffusion-weighted imaging
EEG: Electroencephalography
EPA: Expert panel on Alzheimer
FDG-PET: Fluorodeoxyglucose positron emission tomography
FLAIR: Fluid-attenuated inversion recovery
FSE: Fast spin-echo
GP: General practitioner
GPCog: General practitioner assessment of cognition
GRE: Gradient echo
MCI: Mild cognitive impairment
MMSE: Mini mental state examination
MoCA: Montreal cognitive sssessment
MRI: Magnetic resonance imaging
PC-FI: Primary care frailty index
PDTA: Diagnostic and therapeutic care pathway
PET: Positron emission tomography
PHQ-9: Patient health questionnaire-9
SWI: Susceptibility-weighted imaging
